# Characteristics of chlamydia-like organisms pathogenic to fish

**DOI:** 10.1007/s13353-015-0303-8

**Published:** 2015-07-10

**Authors:** Małgorzata Pawlikowska-Warych, Wiesław Deptuła

**Affiliations:** Department of Microbiology, Faculty of Biology, University of Szczecin, Felczaka 3c, 71-412 Szczecin, Poland

**Keywords:** Fish, Chlamydia-like organisms, Epitheliocystis

## Abstract

Bacteria from the *Chlamydiales* order have been long known, especially as pathogenic bacteria to humans and many animal species, principally including birds and mammals. But for slightly over 20 years, they have been identified in the aquatic environment as endosymbionts of amoebas and sea worms. For several years, they have also been recorded as a cause of diseases among fish, causing respiratory system infections in the form of epitheliocystis of the gill. At present, 11 chlamydia-like organisms pathogenic to fish have been described, including nine new ones, classified into six families, four of which are already known (*Parachlamydiaceae*, *Rhabdochlamydiaceae*, *Candidatus* Parilichlamydiaceae, *Candidatus* Clavichlamydiaceae) and two newly created families, namely *Candidatus* Actinochlamydiaceae and *Candidatus* Parilichlamydiaceae. This paper characterises 11 chlamydia-like organisms, as well as seven isolates not classified into families, which are pathogenic to fish, presenting their genetical properties allowing for their classification, as well as morphological properties and diseases caused.

## Introduction

Diseases among fish are caused by many pathogens, including bacteria (Mitchell and Rodger 2011; Joh et al. 2013), viruses (Pierce and Stepien 2012), fungi (Rodriguez-Tovar et al. 2011; Steckler et al. 2014; Yu et al. 2014) and parasites (Mansell et al. 2005; Steinum et al. 2010; Mitchell and Rodger 2011; Karlsbakk et al. 2013). Among bacterial pathogens, the most frequently listed include *Aeromonas salmonicida* (Dallaire-Dufresne et al. 2014), *Flavobacterium* sp. (Zamora et al. 2013), *Vibrio alginolyticus* (Samad et al. 2014), as well as microbes recognised as zoonotic pathogens, such as *Vibrio vulnificus*, *Edwardsiella tarda*, *Streptococcus iniae*, *Mycobacterium marinum* (Haenen et al. 2013), as well as *Citrobacter freundii* and *Aeromonas hydrophila* (Joh et al. 2013). Also, recently, as one of the causes of bacterial diseases in fish indicated bacteria from the *Chlamydiales* order, *Candidatus* Piscichlamydiaceae and *Candidatus* Clavichlamydiaceae (Table 1), which contribute to skin and gill epithelial cysts, so-called epitheliocystis (Nowak and LaPatra 2006; Stride et al. 2014). It must be added that the contribution of bacteria from the *Chlamydiales* order, as the cause for fish diseases, was doubted due to prior lack of association between those pathogens and the aquatic environment. In recent years, however, it has been proved that new species have been described among those bacteria, named chlamydia-like organisms (CLOs), which are generally found in the aquatic environment, including as endosymbionts of amoebas (Corsaro and Venditti 2009) and sea worms of the *Xenoturbella* genus (Israelsson 2007; Kjeldsen et al. 2010), as a component of sponge bacterial flora (Zhu et al. 2008) and as an element of the aquatic environment without a specific host (Thomas et al. 2006; Corsaro et al. 2009). It must be added that, among the previously known CLOs prevalent in the aquatic environment, such as *Estrella lausannensis* and *Parachlamydia acanthamoebae*, as well as *Waddlia chondrophila* isolated from cattle (Table 1), it was evidenced that they possess the ability to infect fish cell lines, whereas the greatest lesions in those cultures were caused by *Waddlia chondrophila* (Kebbi-Beghadadi et al. 2011).

**Table 1:** Current taxonomy of the *Chlamydiales* order (Martel et al. 2012, 2013; Pawlikowska and Deptuła 2013; Vorimore et al. 2012; Sachse et al. 2014)

Family I	Chlamydiaceae
Genus	*Chlamydia* (*C.*)
Species	*C. trachomatis*
Species	*C. suis*
Species	*C. muridarum*
Species	*C. psittaci*
Species	*C. abortus*
Species	*C. felis*
Species	*C. caviae*
Species	*C. pecorum*
Species	*C. pneumoniae*
Species	*C. ibidis*
Species	*C. gallinacea*
Species	*C. avium*
Species	*Candidatus* Amphibiichlamydia ranarum
Species	*Candidatus* Amphibiichlamydia salamandrae
Family II	Parachlamydiaceae
Genus	*Parachlamydia* (*P.*)
Species	*P. acanthamoebae*
Genus	*Neochlamydia* (*N.*)
Species	*N. hartmannellae*
Genus	*Protochlamydia* (*Pr.*)
Species	*Pr. amoebophila*
Species	*Pr. neagreliophila*
Species	*Candidatus* Metachlamydia lacustris
Family III	Simkaniaceae
Genus	*Simkania* (*S.*)
Species	*S. negevensis*
Genus	*Candidatus* Fritschea
Species	*Candidatus* Fritschea bemisiae
Species	*Candidatus* Fritschea eriococci
Family IV	Rhabdochlamydiaceae
Genus	*Rhabdochlamydia* (*R.*)
Species	*R. porcellionis*
Species	*R. crassificans*
Family V	Waddliaceae
Genus	*Waddlia* (*W.*)
Species	*W. chondrophila*
Species	*W. malaysiensis*
Family VI	*Candidatus* Piscichlamydiaceae
Genus	*Candidatus* Piscichlamydia
Species	*Candidatus* Piscichlamydia salmonis
Family VII	*Candidatus* Clavichlamydiaceae
Genus	*Candidatus* Clavichlamydia
Species	*Candidatus* Clavichlamydia salmonicola
Family VIII	Criblamydiaceae
Genus	*Criblamydia*
Species	*Criblamydia sequanensis*
Species	*Estrella lausannensis*

These specific and characteristic lesions, specified as epitheliocystis in fish, were recorded for the first time among carps (*Cyprinus carpio* L.) in 1920, although the name occurred in the 1960s in reference to gill cysts described in bluegill (*Lepomis macrochirus*) (Hoffman et al. 1969). In many cases, epitheliocystis in fish have a mild form, but can also be manifested by such lesions as hypertrophy and infections of epithelial tissues and fish growth inhibition (Lewis et al. 1992; Crespo et al. 1999). Also described were cases where these changes could spread to other tissues of fish, such as gill lamellae, causing respiratory disorders and death of fish (Meijer et al. 2006; Draghi et al. 2007). It was also described that, in fish populations with a high mortality rate (even up to 100 %), those lesions can be manifested with exophthalmoses and lens prolapse, opacity of the cornea and blindness, as well as skin ulcers and backbone abnormality (Syasina et al. 2004). Typical lesions in the form of epitheliocystis as a result of CLOs infection have been described in farmed fish, including barramundi (*Lates calcarifer*) (Stride et al. 2013c), white sturgeon (*Acipenser transmontanus*) (Groff et al. 1996), silver perch (*Bidyanus bidyanus*) (Frances et al. 1997), Atlantic salmon (*Salmo salar*) (Nylund et al. 1998), red seabream (*Pagrus major*) (Syasina et al. 2004), carp (*Cyprinus carpio*) (Kim et al. 2005; Nowak and LaPatra 2006) and yellowtail amberjack (*Seriola lalandi*) (Mansell et al. 2005). Also, in bluestripe snapper (*Lutjanus kasmira*), the newly described CLO was identified, *Candidatus* Renichlamydia lutjani (Table 2), causing cysts in the kidneys and spleen, but not causing them in gills (Work et al. 2003). Moreover, in farmed salmon (*Salmo salar*), it was evidenced that aetiological factors for epitheliocystis are betaproteobacteria *Candidatus* Branchiomonas cysticola (Toenshoff et al. 2012) and *Tenacibaculum maritimum* (Rodger et al. 2011). Also, aetiological factors of epitheliocystis are CLOs known also as epitheliocyst-like organisms (ELOs), not assigned into families, which are characterised with a typical developmental cycle of *Chlamydiales* (Rockey and Matsumoto 2000). They have been identified in epitheliocystis in, among others, white sturgeon (*Acipenser transmontanus*) (Groff et al. 1996), red seabream (*Spartus aurata*) (Crespo et al. 1999), carp (*Cyprinus carpio*) (Kim et al. 2005), sharpsnout seabream (*Diplodus puntazzo*) (Katharios et al. 2008), leopard shark (*Triakis semifasciata*) (Polkinghorne et al. 2010) and spotted eagle ray (*Aetobatus narinari*) (Camus et al. 2013).

**Table 2:** *Chlamydiales* pathogenic to fish (Draghi et al. 2004, 2007; Meijer et al. 2006; Karlsen et al. 2008; Polkinghorne et al. 2010; Corsaro and Work 2012; Kumar et al. 2012; Camus et al. 2013; Fehr et al. 2013; Pawlikowska and Deptuła 2013; Stride et al. 2013a, b, c; Steigen et al. 2013, 2015; Nylund et al. 2015)

Family I	Chlamydiaceae
Family II	Parachlamydiaceae
	CRG18
Family III	Simkaniaceae
Species	*Candidatus* Syngnamydia venezia
Species	*Candidatus* Syngnamydia salmonis
Family IV	*Rhabdochlamydiaceae*
Species	*Candidatus* Renichlamydia lutjani
	CRG20
Family V	Waddliaceae
Family VI	*Candidatus* Piscichlamydiaceae
Genus	*Candidatus* Piscichlamydia
Species	*Candidatus* Piscichlamydia salmonis
Species	*Candidatus* Piscichlamydia cyprinis
Family VII	*Candidatus* Clavichlamydiaceae
Genus	*Candidatus* Clavichlamydia
Species	*Candidatus* Clavichlamydia salmonicola
Family VIII	Criblamydiaceae
Family IX	*Candidatus* Actinochlamydiaceae
Species	*Candidatus* Actinochlamydia clariae
Family X	*Candidatus* Parilichlamydiaceae
Species	*Candidatus* Parilichlamydia carangidicola
Species	*Candidatus* Similichlamydia latridicola
Species	*Candidatus* Similichlamydia laticola
Species	*Candidatus* Similichlamydia labri
Family incerta sedis	UFC1
	UGA1
	WB13
	WB258
	CRG98

## CLOs infecting fish

So far, two bacteria (*Candidatus* Piscichlamydia salmonis and *Candidatus* Clavichlamydia salmonicola) from the *Chlamydiales* order (Table 1) have been described, which cause pathogenic lesions in fish. At present, nine new pathogenic CLOs to fish have been identified, causing lesions in the form of gill epitheliocystis. It should be stated that the CLOs which are reported first as a cause of specific cysts in fish was the aforementioned *Candidatus* Piscichlamydia salmonis from the *Candidatus* Piscichlamydiaceae family (Table 1), isolated from farmed Atlantic salmon (*Salmo salar*) (Draghi et al. 2004). At present, it has been evidenced that the bacteria are characterised with a typical developmental cycle of *Chlamydiales* with three forms [elementary body (EB), reticular body (RB) and intermediate body (IB)], while their genetic similarity to the bacteria from the Parachlamydiaceae family amounts to 81–82 % and 80 % to the bacteria from the *Chlamydia* genus (Table 1) (Draghi et al. 2004). Apart from Atlantic salmon, these bacterium have also been identified in gill cysts in farmed Arctic charr (*Salvelinus alpinus*) in the USA and Canada (Draghi et al. 2010) and in wild brown trout (*Salmo trutta*) in Swiss rivers (Schmidt-Postahaus et al. 2012), whereas in gill epitheliocystis of those fishes, genetical material of *Candidatus* Clavichlamydia salmonicola from the *Candidatus* Clavichlamydiaceae family was found (Table 1) (Schmidt-Postahaus et al. 2012).

Another previously known bacterium causing gill cysts is the aforementioned *Candidatus* Clavichlamydia salmonicola from the *Candidatus* Clavichlamydiaceae family (Table 1), isolated from gill cysts of Norwegian salmon and from gills of Atlantic salmon farmed in Ireland, as well as from wild salmon where gill cysts and symptoms from the respiratory system were recorded (Karlsen et al. 2008). The studies based on their 16S rRNA gene showed over 90 % similarity to the bacteria from the *Chlamydiales* order, while phylogenetic analysis placed them in the *Chlamydiaceae* family and ECLVII (environmental chlamydial lineage) (Table 1) (Karlsen et al. 2008). Despite those facts, the revealed similarity between *Candidatus* Clavichlamydia salmonicola and *Candidatus* Piscichlamydia salmonis, at the level of 81 %, those bacteria formed a new family, *Candidatus* Clavichlamydiaceae, within the *Chlamydiales* order (Table 2) (Karlsen et al. 2008).

The next bacterium among *Chlamydiales*, presently described, which causes pathogenic lesions in fish is *Candidatus* Actinochlamydia clariae (Table 2). This bacterium was detected in gill epitheliocystis in juvenile forms of the African sharptooth catfish (*Clarias gariepinus*), farmed in Uganda (Steigen et al. 2013), where respiratory symptoms and a high mortality rate were recorded. In histopathological studies, in fishes’ gills, hyperplasia of infected cells was detected, with inclusions inside surrounded with a double membrane. Furthermore, in the infected cell structure, apart from the shifting of cellular organelle to the sides, tunnels and tubular structures have been observed, radially stretching from the inclusion membrane, probably built of protein (Steigen et al. 2013). The forms were compared to actin which gives shape to the cell, and—as in this case—allows for opening the cellular membrane and entry into the cytosol of the neighbouring cell (Steigen et al. 2013). In these bacterium, three morphological forms in their developmental cycle were also found, namely EB, RB and IB. Phylogenetic analysis based on the obtained partial sequence of the 16S rRNA gene of those bacteria, compared to analogical sequences of the representatives of all eight families from the *Chlamydiales* order (Table 1), revealed similarity from 80.5 % to 86.3 %, whereas the highest similarity was recorded between the sequence obtained and *Candidatus* Piscichlamydia salmonis, although morphologically, two such bacteria are not similar. Hence, on the basis of phylogenetic studies, considering the criteria proposed by Everett et al. (1999), establishment of a new, ninth Actinochlamydiaceae family was declared in the *Chlamydiales* order (Table 2) (Steigen et al. 2013).

Another CLO pathogenic to fish is *Candidatus* Parilichlamydia carangidicola (Table 2), isolated from gill cysts of yellowtail amberjack (*Seriola lalandi*) living near the South Australian coast (Stride et al. 2013a). Microscopic analyses of fishes’ gills revealed elongated RBs, spherical IBs and no EBs in their cysts. Genetic analysis on the basis of a fragment of 16S rRNA revealed 87 % similarity to the analogical sequence of *Candidatus* Piscichlamydia salmonis, hence the name for the species, *Candidatus* Parilichlamydia carangidicola, was proposed, as well as the name for the new, tenth family within the *Chlamydiales* order: *Candidatus* Parilichlamydiaceae (Table 2) (Stride et al. 2013a).

Another currently described factor causing epithelial cysts in fish is *Candidatus* Similichlamydia latridicola (Table 2), found in gill epithelial cysts in striped trumpeter (*Latris lineata*) (Stride et al. 2013b). Molecular studies on this bacterium on the basis of the 16S rRNA gene revealed a similarity at the level of 93.7–94 % to the sequence of the *Candidatus* Parilichlamydia carangidicola taxon, and 88 % similarity to *Candidatus* Piscichlamydia salmonis. In turn, phylogenetic analysis revealed that taxons (*Candidatus* Similichlamydia latridicola, *Candidatus* Parilichlamydia carangidicola) form a separate clad; hence, on the basis of the criteria proposed by Everett et al. (1999), it was considered justified to establish a separate, eleventh new family *Candidatus* Parilichlamydiaceae within the *Chlamydiales* order (Table 2).

The other new species is *Candidatus* Similichlamydia laticola (Table 2), which, similarly to *Candidatus* Similichlamydia latridicola, was isolated from epithelial cysts in farmed barramundi (*Lates calcarifer*) in Australia, in various developmental stadium, starting from eggs before hatching, through individuals aged 514 days (Stride et al. 2013c). Histopathological studies revealed that gill cysts in fish are located in gill lamellae, both at the base and at the centre and the edge, are basophil and surrounded with a membrane. In the in situ hybridisation test using specific probes for the *Chlamydiales* order for the 16S rRNA gene, the bacterium responds positively, while phylogenetic analysis revealed that the sequences obtained from all individuals analysed yielded 97.1–97.5 % similarity to the analogical sequence of *Candidatus* Similichlamydia latridicola from the Candidatus *Parilichlamydiaceae* family (Table 2).

Another described factor in the *Chlamydiales* order causing diseases among fish is *Candidatus* Similichlamydia labri, identified in gill epithelial cysts in ballan wrasse (*Labrus bergylta*) in Norway (Steigen et al. 2015), which featured RBs, IBs and EBs. Morphologically, cysts and inclusions with those bacteria resembled the same forms observed for *Candidatus* Actinochlamydia clariae, although actin, characterised for the aforementioned bacterium, was not found here. Genetic studies on the basis of the 16S rRNA sequence revealed 97.1 % similarity to *Candidatus* Similichlamydia latridicola, 94.9 % similarity to *Candidatus* Actinochlamydia clariae and 93.3 % similarity to *Candidatus* Parilichlamydia latridicola. According to the postulates by Everett et al. (1999), it was assigned to the *Candidatus* Parilichlamydiaceae family (Table 2).

The next, also recently described bacterium among CLOs pathogenic to fish is *Candidatus* Syngnamydia venezia (Table 2), isolated from gill cysts of broadnosed pipefish (*Syngnathus typhle*), living in the Venice lagoon (Fehr et al. 2013). The cysts were oval in shape and anchored in gill lamellae, while the histological study revealed intracellular granular material with bacteria sized 1–2 μm. Their allocation to the *Chlamydiales* order was checked using the fluorescence in situ hybridisation (FISH) method with Chls-523 marker. In turn, molecular analysis of their material, based on the 16S rRNA gene, revealed the highest similarity to the analogical sequence of an unnamed isolate obtained from *Xenoturbella westbladi* (96 %) and to *Fritschea eriococci* (94 %) and *Fritschea bemisiae* (94 %), as well as *Simkania negevensis* (93 %) from the Simkaniaceae family (Table 1). Therefore, it was proposed to establish a new genus in the Simkaniaceae family, namely *Candidatus* Syngnamydia venezia (Table 2), a species which is characterised with the lack of two-phase developmental cycle, as no EBs were observed in them, or other infectious forms, but exclusively RB bodies, which are probably capable of initiating the infection (Fehr et al. 2013).

Another described CLO pathogenic to fish is *Candidatus* Syngnamydia salmonis (Table 2), identified in gill cysts of Atlantic salmon (*Salmo salar* L.) farmed in Norway, which recorded a high mortality rate (Nylund et al. 2015). Using an electron microscope, cysts of those fish were found to contain bacteria with a different appearance than previously observed, surrounded with a double membrane. The forms were large, elongated (up to 2.5 μm long) and contained granular (ribosomes) and amorphous material (chromatin), which was deemed to be RBs). In turn, the described smaller rod-shaped forms (0.7–1.0 × 0.4–0.5 μm), with peak disc (cap) and a semi-transparent zone similar to vacuoles underneath, was recognised as EBs. Also, forms sized 500–800 nm were observed, namely IBs. Genetic analysis of 16S rRNA in those bacteria revealed that, in over 90 % (91.7–98.1 %), it conforms to analogical sequences of the representatives of the Simkaniaceae family (Table 1), whereas the highest similarity was recorded in the case of symbiont *Xenoturbella westbladi* (98.1 %) and *Candidatus* Syngnamydia venezia (95.7 %), as well as *Fritschea eriococci* (94.5 %), *Fritschea bemisiae* (94.3 %) and *Simkania negevensis* (91.7 %). Therefore, on the basis of the obtained results, it was determined that the bacterium is grouped together with the still unnamed isolate (SM081012-5S) obtained from corkwing wrasse (*Symphodus melops*) and with the isolate from *Xenoturbella westbladi*, in the Simkaniaceae family (Table 2) (Nylund et al. 2015).

The other new species is *Candidatus* Renichlamydia lutjani (Table 2), which was observed in bluestripe snapper (*Lutjanus kasmira*) (Corsaro and Work 2012). This bacterium was found in the kidneys and spleen of those fish, in inclusions resembling epitheliocystis containing Gram-negative bacteria and stained positive using the Gimenez method. Genetical analysis revealed that 300-bp sequence bp of 16S rRNA is in 88.2–88.7 %, similar to the analogical sequence of the representatives of the Rhabdochlamydiaceae family; thus, the bacterium was assigned to this family (Table 2), although it is also 85–86.7 % identical to the representatives of the Simkaniaceae family (Table 1) (Corsaro and Work 2012).

Another described CLO is *Candidatus* Piscichlamydia cyprinis (Table 2), identified in gill cysts of farmed grass carp (*Ctenopharyngodon idella*), suffering symptoms of the respiratory system (Kumar et al. 2012). In those fish, cysts were placed at the base of gill lamellae, and were filled with granular basophilic material. Genetical analysis of a fragment of the 16S rRNA gene revealed 96 % similarity to the analogical sequence of *Candidatus* Piscichlamydia salmonis, which may point to the fact that both species belong to the *Candidatus* Piscichlamydiaceae family (Table 2).

Another group of CLOs causing pathogenic lesions in fish, also named as ELOs, are bacteria not assigned into families in the *Chlamydiales* order. Those bacteria were observed in white sturgeon (Groff et al. 1996), in gill cysts of which bacteria with three developmental forms (EB, RB and IB) were found, while serological tests proved positive for chlamydial lipopolysaccharide (LPS). Analogical observations using microscope analysis and reactions with chlamydial LPS carried out on gill cysts in red seabream (*Spartus aurata*) farmed near the Spanish coast (Crespo et al. 1999), as well as carp (*Cyprinus carpio*) in South Korea (Kim et al. 2005), also pointed to three forms specific to *Chlamydiales* in lesions, as a result of which those bacteria were classified as CLOs. Similar observations were made in Crete in the larvae of farmed sharpsnout seabream (*Diplodus puntazzo*), among which a high mortality rate was recorded (Katharios et al. 2008) and which had lesions in the form of cysts located principally within the primary fin, skin epithelium and rostrum. Histological studies inside the cysts pointed to the presence of particles determined to be elementary and reticulate bodies, characteristic of *Chlamydiales*, although no cellular response was recorded in them, including cell hypertrophy in the infected tissues (Katharios et al. 2008).

Also in osteochondral fish, such as leopard shark (*Triakis semifasciata*) (Polkinghorne et al. 2010) and spotted eagle ray (*Aetobatus narinari*) (Camus et al. 2013), chlamydia-like factors of epitheliocystis were described. In the case of leopard shark, the UFC1 isolate was described (Table 2), the genetic analysis of which revealed 89 % affinity to the representatives of the *Chlamydiales* order, although it created a separate line in this order (Polkinghorne et al. 2010). In serological studies, the UFC1 isolate did not reveal a positive reaction with chlamydial LPS, while in histopathological studies, it was pointed out that it causes macrophage infiltration in gill epithelial cells, which were in the majority of necrotic cells (Polkinghorne et al. 2010). In turn, in spotted eagle ray, the UGA1 isolate was analysed (Table 2), characterised with the fact of inclusions featuring a uniform pool of elongated cells, with the lack of positive reaction with anti-chlamydial antibodies, whereas genetic analysis of 16S rRNA pointed to 85–89 % similarity to *Candidatus* Piscichlamydia salmonis (Camus et al. 2013). Also, while analysing epithelial cysts of Arctic charr (*Salvelinus alpinus*), bacteria were found, marked as isolates WB13 and WB258 (Table 2), revealing 97–100 % similarity to isolates from the *Neochlamydia* genus (Table 1) (Draghi et al. 2007). The same team (Draghi et al. 2010), while analysing Arctic charr (*Salvelinus alpinus*), apart from identification of the aforementioned *Candidatus* Piscichlamydia salmonis (Table 2) in the material sampled from the cysts, also detected a chlamydia-like sequence (GenBank accession number FJ168566) revealing over 80 % similarity to the analogical sequence (16S rRNA) of the representatives of the *Chlamydiales* order with simultaneous similarity to known chlamydia-like sequences remaining below 92 %. Also, in epithelial cysts from gills of leafy seadragon (*Phycodurus eques*) (CRG20), silver perch (*Bidyanus bidyanus*) (CRG18) and in cysts from gills and skin of barramundi (*Lates calcarifer*) (CRG98), bacteria were observed, the phylogenetic analysis of which evidenced that they belong to families in the *Chlamydiales* order and are grouped as follows, respectively: CRG20 with *R. porcellionis* in the *Rhabdochlamydiaceae* family, CRG18 with endosymbionts of amoebas TUME1 and UWC22 in the *Parachlamydiaceae* family, while CRG98 forms a separate line between lines with *Piscichlamydia salmonis* and *R. porcellionis* (Meijer et al. 2006).

## Conclusion

The presented data indicate that, at present, 11 chlamydia-like organisms (CLOs) pathogenic to fish are known, including nine newly identified, classified into six families (*Parachlamydiaceae*, *Rhabdochlamydiaceae*, *Candidatus* Parilichlamydiaceae, *Candidatus* Clavichlamydiaceae, *Candidatus* Actinochlamydiaceae and *Candidatus* Parilichlamydiaceae), principally on the basis of genetic studies. A common characteristic of those bacteria is the fact of infecting epithelial cells of fishes, prevalence of developmental forms typical of *Chlamydiales* and causing an immune response in the form of macrophage infiltrations, as well as impossibility of their in vitro cultivation. It must be added that it was experimentally evidenced that epithelial cells in fish are also sensitive to infection with CLOs and causing immune response, such as *Waddlia chondrophila* and *Parachlamydia acanthamoebae*, as well as *Estrella lausannensis*, which may constitute a basis for analogical studies, including immunological studies, related to bacteria causing epitheliocystis. Such facts evidence that, despite *Chlamydiales* being known for hundreds of years, there is a continuous need for monitoring them, as currently presented CLOs forms are generally prevalent bacteria in the aquatic environment, which pose a real threat to the life and health of fish, and indirectly possibly also to the health of mammals, including humans.
